# Community voices on health, wellness, and engagement: A qualitative process evaluation of a university extension program

**DOI:** 10.1016/j.dialog.2026.100333

**Published:** 2026-07-29

**Authors:** Aaron A. Funa

**Affiliations:** a College of Health and Science, Sorsogon State University, Sorsogon, Philippines; b Graduate School, Sorsogon State University, Sorsogon, Philippines

**Keywords:** Community wellness, Implementation determinants, Noncommunicable disease prevention, Process evaluation, Service learning, University extension

## Abstract

Noncommunicable diseases demand community programs that are meaningful, feasible, and sustainable. This qualitative process evaluation examined a university-led public health and wellness extension program implemented in Philippine local communities. It documented how participants define health, wellness, and engagement; describe perceived benefits and prevention relevance; identify barriers and facilitators to participation; and articulate improvement priorities. Data were generated from semi-structured interviews with community beneficiaries (*n* = 10) and documentary testimonials from faculty extensionists (*n* = 4) and student extensionists (*n* = 7). Reflexive thematic analysis showed that participants framed health as holistic (physical and mental) and linked wellness to practical routines including stress regulation, self-care, nutrition-related skills, and movement. Engagement reflected belonging and psychological safety that supported sharing and sustained attendance, alongside diffusion of learning to others. Perceived benefits included increased health knowledge, coping and stress relief, practical lifestyle skills, and accessible prevention supports. Barriers included limited slots, scheduling constraints, fatigue, and contextual stressors, while facilitators included continuity, adequate resources, and community cooperation. The study proposes a contextually grounded process model linking activities, participant meanings, perceived benefits, implementation conditions, and improvement priorities to support iterative refinement of community wellness programming in similar university-extension settings.

## Highlights

• Participants defined health as holistic and wellness as doable daily routines.
• Psychological safety and belonging supported sharing, trust, and participation.
• Reported benefits included coping skills, lifestyle practices, and prevention support.
• Capacity, scheduling, fatigue, and stressors constrained program participation.
• A transferable process model linked activities, meanings, benefits, and conditions.

## 1 Introduction

Noncommunicable diseases (NCDs) remain a dominant global health burden, with the World Health Organization (WHO) reporting at least 43 million deaths from NCDs in 2021, representing a large majority of non-pandemic-related deaths worldwide [1]. In the Philippines, the pattern is similarly pressing: WHO's country profile indicates that in 2021, NCDs accounted for about 68% of all deaths, highlighting the continuing need for prevention, health promotion, and sustained community-level action [2]. Alongside NCD risks, preventable conditions shaped by social determinants persist. National nutrition surveillance reports, for instance, show that 23.6% of Filipino children under five were stunted in 2023, signaling chronic constraints on growth and well-being that require locally responsive, equity-oriented solutions [3].

Addressing these challenges requires clarity about the core constructs being promoted and evaluated. Health is commonly anchored in WHO's constitutional definition as “a state of complete physical, mental and social well-being and not merely the absence of disease or infirmity” [4]. Wellness, by contrast, is often framed as an active, holistic, and developmental process that integrates multiple dimensions of functioning and everyday practice, extending beyond clinical status to how people live, cope, and thrive [5], [6]. In community settings, these distinctions matter because programs may succeed in delivering activities yet still fall short if community members do not experience changes as meaningful, feasible, or culturally fitting.

A third construct, community engagement, is central to how public health and wellness initiatives become sustainable. *Principles of Community Engagement* defines it as “…the process of working collaboratively with groups of people who are affiliated by geographic proximity, special interests, or similar situations with respect to issues affecting their well-being…” ([7], p. 7). Engagement is more than a participation count; it reflects relationship-building, trust, shared decision-making, and local ownership, which shape adoption and continuation of health-promoting behaviors. These ideas align with socioecological and empowerment-oriented perspectives that locate health behavior within interacting levels of influence (intrapersonal to policy) and emphasize community capacity and control as part of health promotion practice [8], [9]. In this study, the socioecological lens guides how barriers and enabling conditions are organized across levels (individual, interpersonal, community, and policy), while the empowerment lens guides interpretation of narratives related to voice, shared decision-making, and perceived control that support sustained engagement.

Within the Philippine context, national directions underscore health promotion and multi-stakeholder action to improve population health and reduce inequities across the life course [10]. These priorities are reinforced by national development planning that highlights human development goals, including better nutrition and stronger social services, which require coordinated action beyond the health sector alone [11]. At the global level, such efforts are consistent with the Sustainable Development Goals, particularly SDG 3 (Good Health and Well-being), which calls for strengthened prevention and more inclusive pathways to health improvement [12].

In 2024, Sorsogon State University (SorSU), in collaboration with DOH Bicol, introduced Program SYNAPSE (Synergistic Yields Network for Advancing Public Health and Scientific Expertise) as a multiyear public health and wellness extension initiative [13]. The program primarily serves community residents in partner barangays through nutrition and diet education, physical activity, mental health and stress-management sessions, self-care services, blood pressure monitoring, and community-based health education. However, the available documentation for Program SYNAPSE primarily describes its objectives and activities; it does not explain how community members interpret health and wellness, which program components they perceive as beneficial and feasible, or what conditions influence participation, trust, and sustained engagement. This constitutes an empirical gap in understanding the program from the perspectives of its intended beneficiaries. It also reflects a methodological gap because activity counts and attendance records cannot explain how local context, relationships, and implementation conditions shape participants' experiences of a university-led community wellness program.

This study addresses these gaps through a qualitative process evaluation centered on participant and stakeholder accounts. Consistent with process-evaluation guidance that emphasizes mechanisms and context [14] and qualitative inquiry that treats experience and meaning-making as evidence [15], the study examines participant-defined meanings, perceived benefits, barriers and facilitators, and recommendations for program refinement. Reflexive thematic analysis supports a systematic interpretation of these accounts while preserving their contextual character and connection to participant perspectives [16], [17].

Guided by these considerations, this study examines how community members and stakeholders involved in the SYNAPSE Extension Program understand and experience health, wellness, and community engagement. The study contributes knowledge in three ways. Empirically, it provides participant-centered evidence on how a Philippine university-led community wellness extension program is interpreted and experienced. Conceptually, it integrates program activities, participant meanings, perceived benefits, implementation conditions, and community-informed priorities into a contextually grounded process model. Practically, it translates community accounts into actionable directions for improving program design, delivery, access, and partnership sustainability. Thus, rather than estimating program impact, the study explains how program value is constructed and shaped within a specific implementation context. Specifically, it seeks to (RO1) document how community members and stakeholders define health, wellness, and engagement within their everyday realities; (RO2) describe perceived benefits, meaning, and relevance of extension activities in relation to health and wellness; (RO3) identify barriers and facilitators influencing participation, trust, and sustained engagement; and (RO4) synthesize community-informed recommendations for refining program design, delivery mechanisms, and partnership strategies.

## 2 Methodology

### 2.1 Research design

This study employed a qualitative process evaluation design to examine how community beneficiaries and extensionists interpreted and experienced the implementation of Program SYNAPSE [14], [15]. Data generation relied primarily on semi-structured individual interviews with community beneficiaries, using an open-ended prompt about their SYNAPSE experience followed by probes to clarify meanings and elicit concrete examples, consistent with established qualitative interviewing practices [18], [19].

In addition, written narrative testimonials from faculty and student extensionists were included as documentary qualitative data because they provided implementation perspectives that beneficiary interviews could not fully capture, including observations of facilitation, delivery conditions, participation dynamics, and operational constraints. Their inclusion enabled comparison between recipient and implementer perspectives and supported the identification of convergence, divergence, and contextual explanations across data sources [20], [21]. The testimonials were not treated as substitutes for, or as equivalent to, beneficiary interviews. Beneficiary accounts remained the primary source of participant-defined meanings and perceived benefits, whereas extensionist accounts were used to elaborate and triangulate implementation processes. This source-specific distinction helped prevent implementer perspectives from overshadowing community voices. Analytically, the study used within-case summaries to retain the coherence of each participant account, followed by reflexive thematic analysis to develop cross-participant patterns relevant to program implementation and sustainability [16], [17], [22]. The study is reported in accordance with the Consolidated Criteria for Reporting Qualitative Research (COREQ) to strengthen transparency and reporting quality [23].

### 2.2 Researcher positionality and reflexivity

In qualitative research, the researcher serves as the primary instrument of interpretation [15]. In this study, the researcher was a male university extensionist, the initiator and leader of Program SYNAPSE, and had prior experience in qualitative research. This positioning provided familiarity with the program's goals, implementation processes, and community setting, which supported contextual sensitivity during interpretation. At the same time, the researcher recognized that close involvement with the program could shape assumptions about its value, delivery, and outcomes.

To reduce the direct influence of the program leader during data generation, semi-structured interviews were conducted by five members of the SYNAPSE program using a common interview guide. However, because these interviewers were also affiliated with the program, their insider position was likewise recognized as a possible source of influence rather than a complete safeguard against bias. Potential power dynamics and prior familiarity were addressed by emphasizing voluntary participation, confidentiality, and the right to withdraw without penalty, and by encouraging participants to share both favorable and challenging experiences. Reflexivity was operationalized through memoing, attention to discrepant accounts, comparison across beneficiary and extensionist perspectives, and periodic analytic debriefing to examine how prior assumptions and program involvement may have shaped interpretation.

### 2.3 Intervention description

Program SYNAPSE is a multiyear, university-led public health and wellness extension initiative implemented from 2024 to 2027. The program remained ongoing at the time of this evaluation. The implementation examined in this study comprised six face-to-face activity days delivered as two three-day, site-based series rather than through a fixed weekly schedule. Days 1 to 3 were conducted in Barangay Salog and focused on diet, nutrition, physical activity, and prevention-oriented health practices. Days 4 to 6 were conducted in Barangay Sampaloc and focused on mental health, stress management, and psychosocial wellness.

Activities across the six program days included nutrition education, diet- and exercise-related activities, mental health promotion, breathing and stress-management exercises, community video presentations, blood pressure monitoring, and onsite self-care services such as massage, haircut, manicure, and pedicure. The attendance records documented approximately 40 participant attendances across the three activity days in Barangay Salog and 155 participant attendances across the three activity days in Barangay Sampaloc. These figures corresponded to an average attendance of approximately 13 participants per activity day in Barangay Salog and 52 participants per activity day in Barangay Sampaloc.

Approximately 36 teaching and non-teaching personnel and 40 student extensionists supported program implementation across the two sites. Faculty and staff served as session facilitators, health and wellness practitioners, counselors, and program coordinators, while student extensionists assisted with activity delivery and participant engagement. Two faculty extensionists held dual roles as health and wellness practitioners and program implementers, and this overlap was considered when interpreting their accounts.

### 2.4 Sampling and context

Purposive sampling was used to recruit information-rich participants and documentary sources that could directly inform how SYNAPSE was experienced, implemented, and refined [24]. Inclusion criteria for community beneficiaries were: (a) direct participation in at least one SYNAPSE activity in Barangay Salog or Barangay Sampaloc, (b) willingness to share experience-based accounts of health, wellness, engagement, perceived benefits, barriers, or recommendations, and (c) provision of informed consent. Inclusion criteria for extensionist accounts were: (a) direct involvement in program delivery, facilitation, counseling, or coordination, and (b) submission of a written narrative testimonial or reflection relevant to implementation processes and participant engagement.

The final qualitative dataset comprised 21 sources: 10 semi-structured interviews with community beneficiaries (P01-P10) and 11 written narrative testimonials or reflections from implementers, including 4 faculty extensionists (FE01-FE04) and 7 student extensionists (SE01-SE07). The five interviewed beneficiaries coded P01–P05 participated in the Days 1 to 3 activities conducted in Barangay Salog, while the five beneficiaries coded P06–P10 participated in the Days 4 to 6 activities conducted in Barangay Sampaloc. The demographic characteristics and roles of all participants and extensionists are presented in Table 1.

**Table 1:** Participant demographic profile.

Stakeholder group	Code	Age	Sex	Role / descriptor
Participants (Community beneficiaries)	P01	38	Female	Community participant; mother
	P02	40	Female	Community participant; mother/caregiver
	P03	48	Female	Barangay Health Worker; women's group president; mother
	P04	30	Female	Community participant; mother
	P05	38	Female	Community participant; mother
	P06	46	Female	Community participant; mother
	P07	45	Female	Community participant; mother
	P08	42	Female	Community participant; mother
	P09	52	Female	Community participant; mother/caregiver
	P10	32	Female	Community participant; mother
Faculty extensionist	FE01	38	Female	Facilitator; mother
	FE02	45	Female	Facilitator; mother
	FE03	29	Female	Budget and implementation support
	FE04	26	Male	Speaker; professional counselor
Student extensionists	SE01	19	Female	Student extensionist; midwifery student
	SE02	20	Male	Student extensionist; midwifery student
	SE03	20	Female	Student extensionist; midwifery student
	SE04	19	Female	Student extensionist; midwifery student
	SE05	19	Female	Student extensionist; midwifery student
	SE06	20	Female	Student extensionist; midwifery student
	SE07	25	Female	Student extensionist; midwifery student

As shown in Table 1, the community beneficiary group consisted entirely of female adult participants aged 30 to 52 years, most of whom were mothers. One participant held community-linked leadership roles as a Barangay Health Worker and women's group president. The faculty extensionist group included four adults aged 26 to 45 years, comprising three females and one male, with roles in facilitation, counseling, and implementation support. The student extensionist group consisted of seven midwifery students aged 19 to 25 years, including six females and one male.

Recruitment and interviewing proceeded iteratively until narrative sufficiency was reached. After each batch of interviews, the author reviewed the transcript summaries, analytic memos, and emerging codes against the four research objectives. Narrative sufficiency was considered achieved when the later interviews repeated previously identified meanings, perceived benefits, barriers and facilitators, and recommendations without generating new substantive codes or requiring modification of the emerging thematic structure. The determination was therefore based on the adequacy and redundancy of information relevant to the research objectives rather than on a predetermined sample size [25], [26], [27]. This determination applied to the community beneficiary interviews, while the documentary testimonials were analyzed separately to triangulate implementation perspectives. To protect privacy, all excerpts were de-identified and are reported using participant codes.

### 2.5 Data collection

Data were generated through semi-structured, on-site interviews conducted by the five program members identified in the Researcher Positionality and Reflexivity section. Interviews began with an open-ended invitation to share experiences and learnings from the SYNAPSE activities, followed by probing questions to clarify meaning and elicit concrete examples [18], [19]. Several interviews were conducted during or immediately after the culminating session, which included community video presentations and onsite self-care services. Interviews were audio-recorded with written informed consent and transcribed verbatim. Where necessary, Bikol or Filipino excerpts were translated into English while retaining their original meaning [28], [29]. Interviews were conducted individually when feasible, although a companion was present in some cases at the participant's request. Written narrative testimonials or reflections from faculty and student extensionists were collected after the activities as documentary qualitative data; their complementary purpose and source-specific analytic role are explained in the Research Design section [20], [21].

### 2.6 Data analysis

Audio-recorded community interviews were transcribed verbatim. Written testimonials from faculty and student extensionists were retained in their original form and analyzed as documentary qualitative data alongside the interview transcripts [20]. Analysis proceeded in two linked phases to preserve each account while generating cross-participant themes relevant to program improvement [14], [22]. First, the researcher conducted repeated readings of each transcript and documentary account and produced within-case summaries. Community beneficiary accounts were examined primarily for participant-defined meanings, perceived benefits, barriers, and recommendations from the recipient perspective. Faculty extensionist accounts were examined primarily for facilitation, counseling, implementation conditions, and observations of participant response. Student extensionist accounts were examined primarily for delivery experiences, participation dynamics, and operational or engagement-related observations.

Second, reflexive thematic analysis was conducted across the full dataset using Braun and Clarke's procedure [16], [17]. Coding was primarily inductive and semantic, with attention to in-vivo phrasing where appropriate to preserve fidelity to participant language [16]. During theme development, convergence and divergence across stakeholder groups were examined explicitly so that community beneficiary perspectives were not collapsed into implementer interpretations. Theme development was conducted by the researcher through iterative review of code-to-data fit and systematic memo writing to document analytic decisions and revisions across cycles of coding and theme refinement [17], [22].

### 2.7 Trustworthiness

Credibility was supported through triangulation of interview transcripts and implementer testimonials or reflections, and through member checking with six participants across stakeholder groups to confirm the accuracy of interpreted meanings [30]. Consistency in data generation was supported through the use of a common semi-structured interview guide across the five interviewers. An audit trail was maintained using dated versions of the interview guide, de-identified transcripts, code or theme memos, and decision notes. Reflexive memoing, attention to discrepant accounts, and periodic analytic debriefing during analysis helped surface assumptions, examine possible influence of researcher and interviewer affiliation with the program, and keep interpretations grounded in participant accounts.

### 2.8 Ethical considerations

Ethical safeguards were applied throughout the study, including voluntary participation, informed consent, confidentiality, and the right to withdraw without penalty. Identifiers were removed from transcripts, and quotations used in reporting were attributed to participant codes to protect privacy. The study remained consistent with the approved scope as process evaluation-centered, avoiding impact claims and positioning findings as implementation- and experience-based evidence intended to strengthen program responsiveness and quality. The study was reviewed and approved by the Sorsogon State University Campus Evaluation and Review Committee (CERCom) (Control No. RDO-SC-2025-38, November 15, 2025).

## 3 Results

### 3.1 RO1: Participant interpretations of health, wellness, and engagement within the context of SYNAPSE activities

RO1 examined how participants interpreted health, wellness, and engagement through their experiences of SYNAPSE activities rather than as decontextualized definitions. Health reflected how participants understood physical and mental well-being in relation to program learning; wellness referred to the self-care and coping practices they associated with everyday functioning; and engagement referred to how they experienced participation, belonging, and involvement in program activities. Under RO1, these meanings clustered into three core constructs with related subthemes, including references to nutrition, diet-related skills, and exercise or movement. Table 2 presents these findings.

**Table 2:** Participant-defined meanings of health, wellness, and engagement with subthemes (RO1).

Core construct	Subtheme	Representative verbatim excerpt (English translation in parentheses)
Health	Holistic health interpreted through program learning	“Ano, nakahatag man nin ano kaaraman sa pagpisikal, mental” (It [Program SYNAPSE] provided knowledge about physical and mental health) (P10)
	Physical health and self-care as part of being healthy	“Nakakatulong po siya para maaraman ang pag-ano sa mental health, sa ano, pisikal tapos Lalo na sa selfcare.” (It helped us understand mental health, physical health, and especially self-care) (P02)
	Nutrition and exercise as health-related content	“workshops, seminars, and activities centered on nutrition, exercise, and mental health.” (SE01)
	Help-seeking and not carrying problems alone	“pag may problema ka palan, diri mo kaipuhan na iano mo lang sa sarili mo… makipag-isturyahan” (When you have a problem, you do not need to keep it to yourself… you can talk about it) (P01)
	Mental health should not be ignored	“wag balewalain ang ano, mental health.” (Do not disregard mental health) (P08)
Wellness	Coping exercises for stress regulation	“Opo, ang pag inhale-exhale, naaapply ko po siya pag grabi ang problema ko…” (Yes, I apply inhale-exhale when the problem is intense) (P01)
	Relief from stress and everyday fatigue	“Nakakahali po ning ano, nakakahali po ng stress bagan ano yung pagod mo sa bahay” (It eases stress, including the tiredness from home) (P10)
	Movement and activity as restorative (exercise-through-participation)	“Masaya kami… nakikita namin ang sarayawan. Napakasaya.” (We were happy… we could watch/see the dancing. We were very happy) (P07)
	Diet-related wellness skills (food preparation)	“knowing that my guidance helped them develop practical cooking skills that they can use throughout their lives.” (FE02)
	Wellness through simple self-care actions	“nagpagupit lang po ako” (I just got a haircut) (P08)
Engagement	Enjoyment and sense of belonging	“Masaya kami kasi nandito kamong lahat… Napakasaya.” (We are happy because all of you are here… We are very happy) (P07)
	Safe space for sharing personal experiences	“created a unique space where participants felt comfortable sharing their personal stories and challenges.” (FE04)
	Consistency and active involvement	“unwavering attendance and active engagement demonstrated their genuine interest in learning.” (FE02)
	Sharing learnings beyond the session	“ipabahagi pa po sa iba.” (Please share it with others) (P08)

#### 3.1.1 Health

Participants framed health as holistic and functional, explicitly linking the physical and mental (“pagpisikal, mental”) and connecting health to self-care as part of day-to-day wellbeing. Health meanings also included lifestyle-oriented content where implementers described activities explicitly centered on nutrition and exercise, indicating that participants and stakeholders recognized these as core components of being “healthy” within the program context. At a deeper level, these accounts suggest that health was not understood primarily as a biomedical state, but as the capacity to cope, seek support, and remain functional within everyday pressures. Health was therefore narrated not only as bodily condition, but as relational resilience, where talking to others and not ignoring mental health were themselves part of being well.

#### 3.1.2 Wellness

Participants defined wellness as actionable practices that restore functioning. This included coping exercises (for example, inhale-exhale) used during intense stress, and the felt outcome of stress relief and reduced fatigue from everyday demands. Importantly for RO1, wellness meanings also reflected nutrition and diet-related practice, described as developing “practical cooking skills,” and exercise or movement expressed through participation in active, dance-related activities. Together, these accounts positioned wellness as both skill-based (what one can do) and experience-based (how one feels after doing it). Latently, wellness was framed less as an abstract ideal and more as something modest, immediate, and doable, suggesting that participants valued interventions that fit daily life rather than those requiring major lifestyle disruption.

#### 3.1.3 Engagement

Participants defined engagement as more than attendance. It included enjoyment, belonging, comfort in sharing personal experiences, sustained participation, and extending learnings to others. Beneficiaries tended to emphasize enjoyment and felt comfort, whereas implementers more often highlighted consistency, safe-space creation, and active participation. This suggests that engagement carried both an experiential meaning for participants and an implementation meaning for facilitators.

### 3.2 RO2: Perceived benefits, meaning, and relevance of the extension program activities in relation to health and wellness

Participants described the extension program as beneficial and relevant to wellness and NCD-prevention practices because it combined health learning, practical lifestyle skills, and feasible wellness supports. Across accounts, these benefits were expressed at the individual, family, and community levels. Table 3 presents the RO2 findings on perceived benefits, meaning, and relevance to NCD prevention and wellness practices.

**Table 3:** Perceived benefits, meaning, and relevance of extension program activities for NCD prevention and wellness practices (RO2).

Domain	Subtheme	Representative verbatim excerpt (English translation in parentheses)
Perceived benefits	Increased health knowledge (mental and physical)	“nagdamo ang knowledge ko… sa mental health” (My knowledge increased… about mental health) (P01)
	Stress reduction through breathing techniques	“ang pag inhale-exhale, naaapply ko po siya pag grabi ang problema ko” (I apply inhale-exhale when the problem is intense) (P01)
	Relaxation and relief from household fatigue	“nakakatanggal siya ng stress… narerelax ka” (It removes stress… you feel relaxed) (P10)
	Practical nutrition-related skills (food preparation)	“my guidance helped them develop practical cooking skills that they can use throughout their lives.” (FE02)
	Lifestyle awareness via nutrition and exercise activities	“activities centered on nutrition, exercise, and mental health.” (SE01)
Meaning	Self-care as a legitimate priority	“kailangan pala magtira ka para sa sarili mo” (You need to set something aside for yourself) (P03)
	Support-seeking instead of carrying problems alone	“hindi mo kailangang sarilinin… Pwede kang makipag-usap sa iba.” (You do not need to keep it to yourself… You can talk to others) (P01)
	Safe space to share experiences	“participants felt comfortable sharing their personal stories and challenges.” (FE04)
Relevance to NCD prevention and wellness practices	Movement-based routines as doable wellness practices	“Inhale, exhale” (P10)
	Self-care services as an accessible entry point	“free massage, haircut, manicure, and pedicure.” (SE02)
	Basic health monitoring as tangible prevention support	“free blood pressure monitoring” (SE07)
	Need for continuity and wider reach	“sana po, tuloy-tuloy… para… ang iba naman… maka avail man… limited” (I hope it continues… so others can also avail… it's limited) (P06)

#### 3.2.1 Perceived benefits: Knowledge, skills, and immediate wellness gains

Participants identified benefits that were both cognitive (learning) and experiential (felt changes). Some emphasized increased understanding of mental health and coping, including the use of breathing strategies during stressful moments: “ang pag inhale-exhale, naaapply ko po siya pag grabi ang problema ko” (I apply inhale-exhale when the problem is intense) (P01). Others framed benefits as immediate stress reduction and relaxation: “nakakatanggal siya ng stress… narerelax ka” (It removes stress… you feel relaxed) (P10). Benefits aligned with NCD-prevention-oriented wellness were also reflected in lifestyle content and skills. Facilitators highlighted nutrition-related learning through “practical cooking skills” (FE02), while student extensionists described activities centered on “nutrition, exercise, and mental health” (SE01). Taken together, these accounts suggest that participants valued benefits that were understandable and immediately usable, rather than distant or purely technical.

#### 3.2.2 Meaning: The extension program as permission, support, and a safe learning space

Participants described what the extension program meant beyond its activities. A recurring meaning involved legitimizing self-care and recognizing personal value: “kailangan pala magtira ka para sa sarili mo” (You need to set something aside for yourself) (P03). Another meaning emphasized support-seeking and communication rather than isolation: “hindi mo kailangang sarilinin… Pwede kang makipag-usap sa iba.” (You do not need to keep it to yourself… You can talk to others) (P01). From the implementers' perspective, the program's meaning included creating a psychologically safe environment: “participants felt comfortable sharing their personal stories and challenges.” (FE04). This indicates a subtle difference in emphasis: beneficiaries tended to describe what the program allowed them to feel or do, whereas implementers more often described the conditions they tried to create. Analytically, this suggests that safety and support functioned not simply as background conditions, but as part of the mechanism through which participants came to see self-care as legitimate and shareable.

#### 3.2.3 Relevance: Accessible but not unlimited pathways for prevention and wellness practices

Participants described the extension program as relevant because it offered practices and supports that felt doable within everyday constraints. Breathing and movement practices were recalled as simple routines (“Inhale, exhale” [P10]), suggesting that participants viewed these as usable coping strategies. Relevance was also tied to accessibility through tangible supports. Student extensionists highlighted free self-care services such as “free massage, haircut, manicure, and pedicure” (SE02), while others noted “free blood pressure monitoring” (SE07), reflecting a practical connection to prevention-oriented awareness. At the same time, relevance was narrated alongside scarcity, as participants' wish for continuity and broader reach (“sana po, tuloy-tuloy… limited” [P06]) implied that usefulness was already recognized, but access remained constrained. This tension suggests that perceived relevance alone did not guarantee equitable uptake.

### 3.3 RO3: Barriers and facilitators influencing participation, trust, and sustained engagement in the extension program

For analytic clarity, trust referred to participants' relational confidence, psychological safety, and willingness to disclose personal experiences within the program. Sustained engagement referred to continued access, attendance, participation, application of learning, and willingness to remain involved or share learning with others. Table 4 identifies which construct was primarily affected by each barrier or facilitator. Psychological safety directly supported trust and could subsequently strengthen sustained engagement, whereas access, scheduling, fatigue, and resource constraints primarily affected sustained engagement rather than trust.

**Table 4:** Barriers and facilitators classified according to their relationship with trust and sustained engagement (RO3).

Domain	Barriers (subthemes with classification and verbatim excerpts)	Facilitators (subthemes with classification and verbatim excerpts)
Program access and capacity	Limited availability of services or slots — Sustained engagement and access “Kasi po baga, limited.” (Because it is limited.) (P06)	Desire for continuity to broaden reach — Sustained engagement “sana po, tuloy-tuloy” (I hope it continues.) (P06)
Scheduling and participation routines	Late arrival as a recurrent constraint — Sustained engagement “Though some of the participants were always late…” (FE02)	Persistence despite constraints — Sustained engagement “…their unwavering attendance and active engagement demonstrated their genuine interest in learning.” (FE02)
Psychosocial readiness and relational conditions	Low initial prioritization of mental health — Readiness for sustained engagement “I discovered that many parents initially didn't prioritize mental health in their families.” (FE04)	Psychological safety — Trust and sustained engagement “created a unique space where participants felt comfortable sharing their personal stories and challenges.” (FE04)
Contextual stressors in daily life	Crisis-related stress — Readiness for sustained engagement “nag-agi po baga ang bagyo… nastress out sinda…” (A typhoon passed… they were stressed…) (P09)	Immediate relief and re-engagement — Sustained engagement “Bagan narelax sinda didi” (They were able to relax here.) (P09)
Tangible supports and implementation capacity	Household fatigue — Readiness for sustained engagement “pagod ka” (You are tired.) (P10)	Accessible wellness entry points — Sustained engagement “provided a free services including a free massage, haircut, manicure, and pedicure.” (SE02) Prevention-oriented monitoring — Sustained engagement “free blood pressure monitoring” (SE07) Adequate implementation resources — Sustained engagement and implementation capacity “the implementation was made easier because… had available and sufficient funding.” (FE03)
Community diffusion and shared ownership	Unequal access — Sustained engagement and reach “limited reach” (P06)	Community cooperation — Sustained engagement and shared ownership “want to cooperate with us.” (SE04) Knowledge-sharing beyond sessions — Sustained engagement and diffusion “ipabahagi pa po sa iba.” (Please share it with others.) (P08)

As shown in Table 4, limited services or slots, scheduling constraints, psychosocial readiness, contextual stress, fatigue, and unequal access primarily affected sustained engagement rather than trust. Trust-related evidence was concentrated in psychological safety, particularly participants' comfort in sharing personal experiences. Psychological safety was classified as affecting both constructs because it directly supported trust and created conditions conducive to continued participation.

Across domains, barriers clustered around capacity limits (limited slots or services), time-related constraints (late arrival), readiness issues (low initial prioritization of mental health), and daily-life stressors (typhoon-related stress, household fatigue). Facilitators clustered around continuity and persistence (desire for the program to continue; steady attendance), psychological safety (comfort sharing stories), tangible supports (self-care services, blood pressure monitoring, sufficient funding), and community diffusion (cooperation and sharing learnings). Analytically, these findings show that engagement was not uniformly enabled or constrained; rather, it was negotiated within everyday conditions. For example, implementers described lateness and low initial prioritization of mental health as challenges, while participants simultaneously described relief, enjoyment, and willingness to continue. This suggests that positive engagement coexisted with partial readiness and uneven access, rather than replacing them.

Importantly, the data also contained less affirmative or constraining cases that complicated a purely positive account of the program. “Limited” access, fatigue, and crisis-related stress indicated that even when participants valued the activities, real-world demands could reduce participation or restrict who benefited. In this sense, the negative or constraining cases did not contradict the program's perceived usefulness, but revealed the conditions under which usefulness could be interrupted, delayed, or unevenly distributed. These patterns reflect a central tension in community-based wellness and NCD-prevention work: programs gain traction when participants feel safe, supported, and able to access concrete services, yet uptake remains vulnerable to practical, psychosocial, and structural constraints.

### 3.4 RO4: Community-informed recommendations for refining program design, delivery mechanisms, and partnership strategies

Under RO4, participants' recommendations focused on improving the extension program's continuity and reach, strengthening community diffusion and shared ownership, refining delivery logistics to match real participation routines, maintaining psychological safety, and preserving an integrated package of nutrition, exercise, mental health, and prevention-oriented monitoring. Table 5 presents the RO4 findings.

**Table 5:** Recommendations and priority directions to strengthen the extension program for health and wellness.

Recommendation domain	Subthemes	Verbatim excerpts
Continuity and expansion of reach	Continue and sustain delivery	“sana po, tuloy-tuloy po ini na paghatag niyo po saamon” (I hope this continues, what you are providing to us.) (P06)
	Repeat activities and offer more sessions	“Sana po maulit gihapon.” (I hope it can be repeated again.) (P06)
	Increase the number of activities	“Sana marami pa kayong activity na tulad po nito.” (I hope you have more activities like this.) (P07)
Access, fairness, and slot allocation	Address limited slots and widen availability	“Kay para po, ang iba naman na diri pa naka avail, maka avail man po. Kasi po baga, limited.” (So that others who have not yet availed can also avail, because it is limited.) (P06)
	Ensure fairness of access	“Oo, limitado lang po maam, di siyempre ang iba man po, para matagan man nin chance.” (Yes, it is limited, so that others can also be given a chance.) (P06)
Community diffusion and shared ownership	Promote sharing of learnings beyond sessions	“ipabahagi pa po sa iba.” (Please share it with others.) (P08)
	Strengthen local champions for dissemination	“pwede ko maishare sa kanila ang mga natutunan ko” (I can share with them what I learned.) (P03)
Delivery logistics and participation routines	Design around late arrival patterns	“Though some of the participants were always late…” (FE02)
	Build on consistent participation despite constraints	“…their unwavering attendance and active engagement demonstrated their genuine interest in learning.” (FE02)
	Continue holding similar community events	“Oo. Masayang masaya.” (Yes. Very, very happy.) (P07)
Psychological safety and readiness-building	Strengthen early prioritization of mental health	“many parents initially didn't prioritize mental health in their families.” (FE04)
	Maintain safe spaces for sharing experiences	“participants felt comfortable sharing their personal stories and challenges.” (FE04)
	Normalize support-seeking and communication	“kapag my problema ka pala hindi mo kailangang sarilinin. Pwede kang makipag-usap sa iba.” (When you have a problem, you don't need to keep it to yourself. You can talk to others.) (P01)
Integrated wellness package for NCD prevention	Keep the lifestyle focus (nutrition and exercise) alongside mental health	“activities centered on nutrition, exercise, and mental health.” (SE01)
	Sustain practical nutrition skills	“practical cooking skills that they can use throughout their lives.” (FE02)
	Maintain accessible wellness services	“provided a free services including a free massage, haircut, manicure, and pedicure.” (SE02)
	Retain prevention-oriented monitoring	“free blood pressure monitoring” (SE07)
Partnership and implementation support	Leverage community willingness to collaborate	“want to cooperate with us.” (SE04)
	Protect implementation capacity through resources	“the implementation was made easier because… had available and sufficient funding.” (FE03)

Recommendations centered on sustaining the program, expanding access beyond “limited” slots, strengthening knowledge-sharing to others, adjusting delivery to real participation routines including lateness, maintaining psychologically safe sessions, and keeping integrated wellness components. More than a list of desired improvements, these recommendations reveal how participants interpreted the program's core value. What participants wanted preserved was not only the content of the activities, but also the conditions that made the program feel accessible, respectful, and worth returning to.

The recommended directions also make visible a tension between appreciation and insufficiency. Participants' generally favorable accounts did not eliminate critique; instead, positive experiences appeared to increase expectations for continuity, fairness, and wider reach. In this sense, recommendations functioned as evidence of engagement rather than simple dissatisfaction. Based on the results, strengthening the extension program can prioritize: (a) additional runs or parallel delivery teams to reduce limited access, (b) structured diffusion via local champions and share-back mechanisms, (c) session designs that remain meaningful despite late arrival, (d) consistent safe-space facilitation, and (e) sustained integration of nutrition, exercise, and monitoring components, backed by stable partnerships and resourcing.

**Fig. 1:**
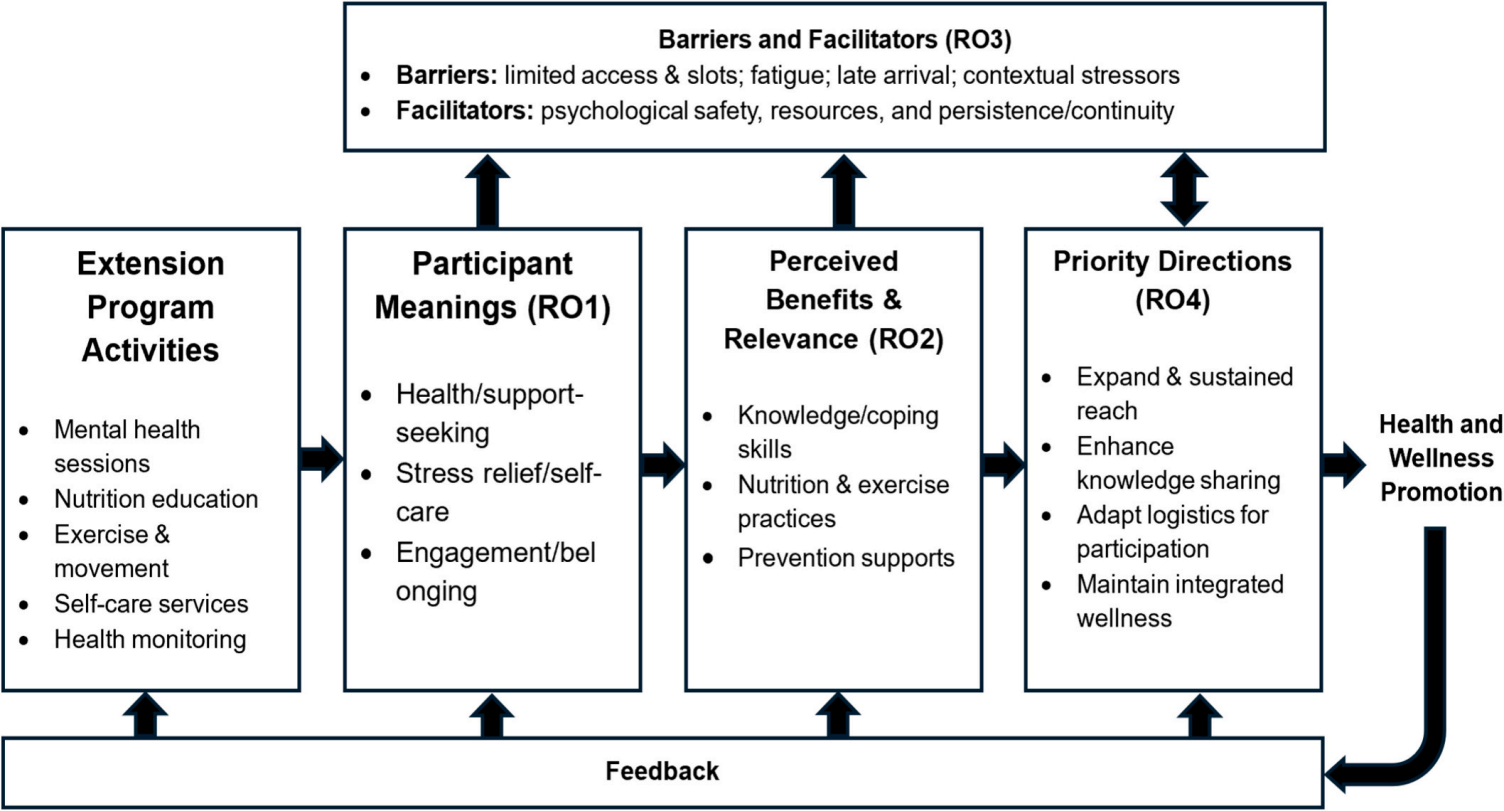
Integrated process model for community wellness programs.

Fig. 1 presents an empirically derived, participant-centered process model explaining how a university-led community wellness program is interpreted, experienced, and refined. The model comprises five linked elements: (1) extension activities; (2) participant-defined meanings of health, wellness, and engagement; (3) perceived benefits and relevance; (4) cross-cutting barriers and facilitators; and (5) community-informed refinement priorities. The directional pathway indicates that activities are not assumed to produce benefits directly. Instead, activities acquire significance through participant meaning-making, while implementation conditions influence whether perceived value becomes feasible and sustained engagement. Community-informed priorities then feed back into subsequent program delivery, making the model iterative rather than linear.

Arrows show the relationships among extension activities, participant meanings (RO1), perceived benefits and relevance (RO2), barriers and facilitators (RO3), priority directions (RO4), and feedback for iterative health and wellness promotion.

The model's novelty lies in how these familiar elements are configured into a single process pathway, rather than in claiming that each element is independently new. Unlike conventional logic models that generally trace inputs, activities, outputs, and outcomes, the proposed model positions participant meaning-making between program delivery and perceived benefits. Unlike broad implementation frameworks that organize determinants across multiple domains, it shows how barriers and facilitators intersect different stages of the participant experience and how community recommendations become feedback for program refinement [14], [31]. The model therefore offers a bounded, contextually grounded explanation of program functioning rather than a universal causal or predictive framework.

The first directional relationship runs from activities to RO1. Program inputs, such as nutrition education, exercise-related participation, mental health sessions, self-care services, and prevention-oriented monitoring, were first interpreted by participants through their own meanings of health, wellness, and engagement. Empirically, this component was grounded in participant accounts that framed health as holistic care and support-seeking, wellness as stress relief, self-care, and doable daily practices, and engagement as belonging, comfort, and shared participation. The next directional relationship runs from RO1 to RO2. This shows that participant meanings shaped how benefits and relevance were perceived. When participants understood the activities as supportive, practical, and personally meaningful, they were more likely to describe benefits such as increased knowledge, coping skills, stress relief, nutrition-related learning, movement-oriented participation, and prevention awareness.

Fig. 1 further positions RO3 as a cross-cutting condition that influences both RO1 and RO2. Barriers and facilitators shaped both the formation of participant meanings and the realization of perceived benefits. Empirically, these included constraints such as limited slots, late arrival, fatigue, and contextual stressors, alongside enabling conditions such as psychological safety, continuity, adequate resources, and community cooperation. The relationship between RO3 and RO4 shows that barriers and facilitators directly informed the recommendations participants proposed. These priorities included expanding reach, improving logistics, strengthening safe-space facilitation, supporting knowledge sharing, and preserving an integrated wellness package. RO4 therefore represents participant-informed refinement priorities derived from the data.

The model then places RO4 as informing health and wellness promotion. In this context, health and wellness promotion is best understood as the applied program-level expression of the empirical findings. Participant-informed priorities are translated into more responsive wellness-promoting action, which in turn feeds back into future activities, participant interpretations, perceived benefits, and further recommendations for improvement. Taken together, these relationships show that the model is iterative rather than linear. Overall, the model is transferable not as a fixed template, but as an analytic guide for similar community health or university-extension settings that rely on face-to-face wellness activities, locally embedded partnerships, participant feedback, and resource-constrained implementation.

## 4 Discussion

This study shows that the value of a community wellness extension program lies not only in activity delivery, but in how those activities are interpreted, supported, and sustained in everyday life. Across RO1 to RO4, participant meanings, perceived benefits, implementation conditions, and improvement priorities functioned as linked process elements rather than isolated findings. This extends process evaluation in community extension settings by showing that uptake depends not simply on attendance or exposure, but on whether activities are experienced as meaningful, feasible, and relationally safe within local realities [14], [31].

Participants' definitions of health and wellness were holistic and practice-oriented, aligning with public health framing that health includes mental and social dimensions and is shaped by everyday functioning and relationships [4]. In RO1, “health” was not limited to physical status; it included mental health, coping, and support-seeking. “Wellness” was articulated as actionable routines (e.g., breathing exercises, self-care behaviors), consistent with wellness theory that views well-being as an active process rather than a static endpoint [5], [6]. “Engagement” was described as belonging and comfort in sharing experiences, highlighting the role of relational conditions in participation. This aligns with community engagement guidance emphasizing trust, inclusion, and partnership quality as prerequisites for sustained involvement and community ownership [32].

RO2 extends these meanings into a prevention-relevant pathway. Participants perceived benefits not only as “learning,” but as practical and repeatable behaviors that can plausibly support NCD risk reduction [1], [33]. The program's relevance was grounded in feasibility: coping routines for stress regulation, nutrition-related skills, movement or exercise participation, and prevention-oriented monitoring were valued because they could be applied in everyday life. This interpretation is consistent with behavior change theory, particularly the COM-B model, which proposes that sustained behavior requires capability (knowledge and skills), opportunity (resources and supportive conditions), and motivation (perceived value and relevance) [34]. In this study, capability was reflected in reported learning and skills (including diet-related and coping skills), opportunity was reflected in accessible entry points (services and monitoring) and a supportive environment, and motivation was reflected in participant-reported meaning and willingness to continue and recommend the program.

The RO3 findings qualify the predominantly affirmative accounts. Limited slots and unequal reach (P06), typhoon-related stress (P09), household fatigue (P10), recurrent lateness (FE02), and low initial prioritization of mental health (FE04) demonstrate that perceived relevance did not ensure equitable access, readiness, or sustained participation. These less affirmative cases did not constitute outright rejection of the program; rather, they revealed conditions under which its perceived benefits could be delayed, interrupted, or unevenly distributed. They also indicated a difference between stakeholder perspectives: beneficiaries generally expressed constraints through brief accounts of scarcity, stress, and fatigue, whereas implementers more directly identified participation and readiness problems. The limited expression of direct dissatisfaction should therefore not be interpreted as uniform approval, particularly because some interviews were conducted by program-affiliated personnel and may have been influenced by courtesy or social-desirability effects.

These cases reinforce a socioecological interpretation of engagement, in which participation is shaped by interpersonal demands, community conditions, and structural constraints rather than individual interest alone [9]. Conversely, psychological safety, continuity, tangible supports, and adequate resources indicate that delivery conditions function as part of the intervention itself [35]. Psychological safety may enable sharing, reflection, and learning [36], while limited access, fatigue, and scheduling difficulties may restrict who benefits and whether engagement can be sustained. These findings are consistent with implementation frameworks that emphasize interactions among intervention characteristics, context, and implementation processes [37].

RO4 recommendations function as participant-driven quality improvement priorities that directly respond to the constraints and enabling conditions identified in RO3. Calls for continuity and expansion address capacity limitations and perceived inequities in access. Suggestions to strengthen knowledge sharing and community cooperation align with diffusion theory, which describes how innovations spread through social networks when they are perceived as advantageous, compatible with local values, and supported by interpersonal communication [38], [39]. Recommendations to maintain a safe space for sharing reinforce engagement theory and underscore that relational quality is part of the program's “active ingredients” [32], [36]. Importantly, participants' emphasis on sustaining an integrated package (mental wellness, nutrition, movement, and monitoring) supports the argument that community members experience health needs as interconnected rather than siloed, which strengthens the case for integrated prevention design in community settings [1], [33].

Fig. 1 synthesizes the findings as a contextually grounded process pathway in which program activities are interpreted through participant meanings, translated into perceived benefits, shaped by implementation conditions, and converted into community-informed priorities. Its contribution lies not in proposing entirely new categories of benefits or barriers, but in explaining how these elements interact within a university-extension setting. This mechanism-oriented interpretation is consistent with complex-intervention theory [31], COM-B [34], psychological safety [36], and diffusion theory [38], [39]. The model consequently offers a bounded analytic explanation of how meaning-making, feasible practices, relational safety, access, and participant-informed adaptation jointly shape the acceptability and sustainability of community wellness programs.

The findings also have practical implications for community wellness and NCD-prevention programming, particularly in settings where NCD burden is high and nutrition constraints persist [1], [2], [3]. Programs may benefit from designing integrated wellness packages that combine coping, nutrition, movement, and basic monitoring while protecting psychological safety as a core delivery feature. Implementation adaptations should also account for real participation routines, including late arrival and fatigue, through modular session designs, recap elements, transparent access procedures, and partnership-driven expansion. Diffusion may be strengthened through structured share-back strategies, local champions, and community cooperation that extend learning beyond direct participants [38], [39].

## 5 Limitations of the study

Several limitations should be considered. As a qualitative process evaluation, the study was designed to explain meanings, perceived benefits, and implementation conditions rather than estimate causal effects or clinical outcomes [14]. Some interviews were conducted during or immediately after program activities, which may have increased social desirability in participant responses. The inclusion of faculty and student extensionist testimonials also strengthened contextual understanding of implementation but may have introduced a more program-affirming perspective into the dataset. In addition, participants may have associated the interview process with the extension initiative and its organizers, which could have reduced willingness to express criticism despite assurances of voluntary participation, confidentiality, and the right to withdraw. Participant accounts may likewise have been shaped by translation and reporting decisions, and some nuance may have been lost in translating excerpts into English [28], [29]. Although participant characteristics are summarized in Table 1, the community beneficiary group consisted entirely of adult female participants, most of whom were mothers, while the extensionist groups were drawn from those directly involved in program implementation. These sample features should be considered in assessing transferability. The findings are therefore most applicable to community health or university-extension programs with similar participant profiles, contextual conditions, resources, and delivery arrangements. Future research may test the model in other communities and include a wider range of participant groups.

## 6 Conclusion and recommendations

In conclusion, the study clarified how participants defined health, wellness, and engagement in practical, relational terms, and showed that the program was perceived as beneficial and relevant to wellness and NCD-prevention practices when activities translated into feasible routines (e.g., stress regulation, nutrition-related skills, movement, and prevention-oriented monitoring). It also identified participation as shaped by capacity limits, scheduling realities, fatigue, and contextual stressors, while psychological safety, continuity, resources, and community cooperation supported engagement and diffusion. Participants recommended sustaining and expanding delivery, improving access and session fit, strengthening knowledge sharing, and maintaining an integrated wellness package. Taken together, the integrated model links activities, meanings, perceived benefits, implementation determinants, and improvement priorities, providing a theoretically grounded framework for iterative refinement and scaling of community-based wellness and NCD-prevention initiatives.

These findings suggest that community wellness extension initiatives should sustain an integrated wellness package that combines mental health support, nutrition and diet skills, movement or exercise activities, and basic prevention monitoring, while ensuring psychological safety as a core delivery feature. Derived from the integrated model, the most critical actionable recommendation for program implementers and policymakers is to institutionalize this integrated package through sustained and expanded delivery, using additional runs or parallel teams, transparent access procedures, and stable partnership support so that limited slots do not restrict equitable participation. Programs may also strengthen community diffusion through structured sharing mechanisms and local champions, and adjust session design to fit participation realities while supporting continuous refinement through feedback. Longitudinal and multi-site designs can examine whether reported wellness practices are maintained over time and identify which delivery adjustments most improve uptake across different community contexts.

## CRediT authorship contribution statement

**Aaron A. Funa:** Writing – review & editing, Writing – original draft, Visualization, Validation, Resources, Project administration, Methodology, Investigation, Formal analysis, Data curation, Conceptualization.

## Declaration of competing interest

The author declare no conflict of interest.
